# Supplementary material for: SOX2 Promotes Invasion in Human Bladder Cancers through MMP2 Upregulation and FOXO1 Downregulation
Source: Int J Mol Sci. 2022 Oct 19;23(20):12532. doi: 10.3390/ijms232012532 (PMC9604292; doi:10.3390/ijms232012532)
Supplement: Supplementary file 1 [file ijms-23-12532-s001.zip › ijms-1927827-supplementary.pdf]

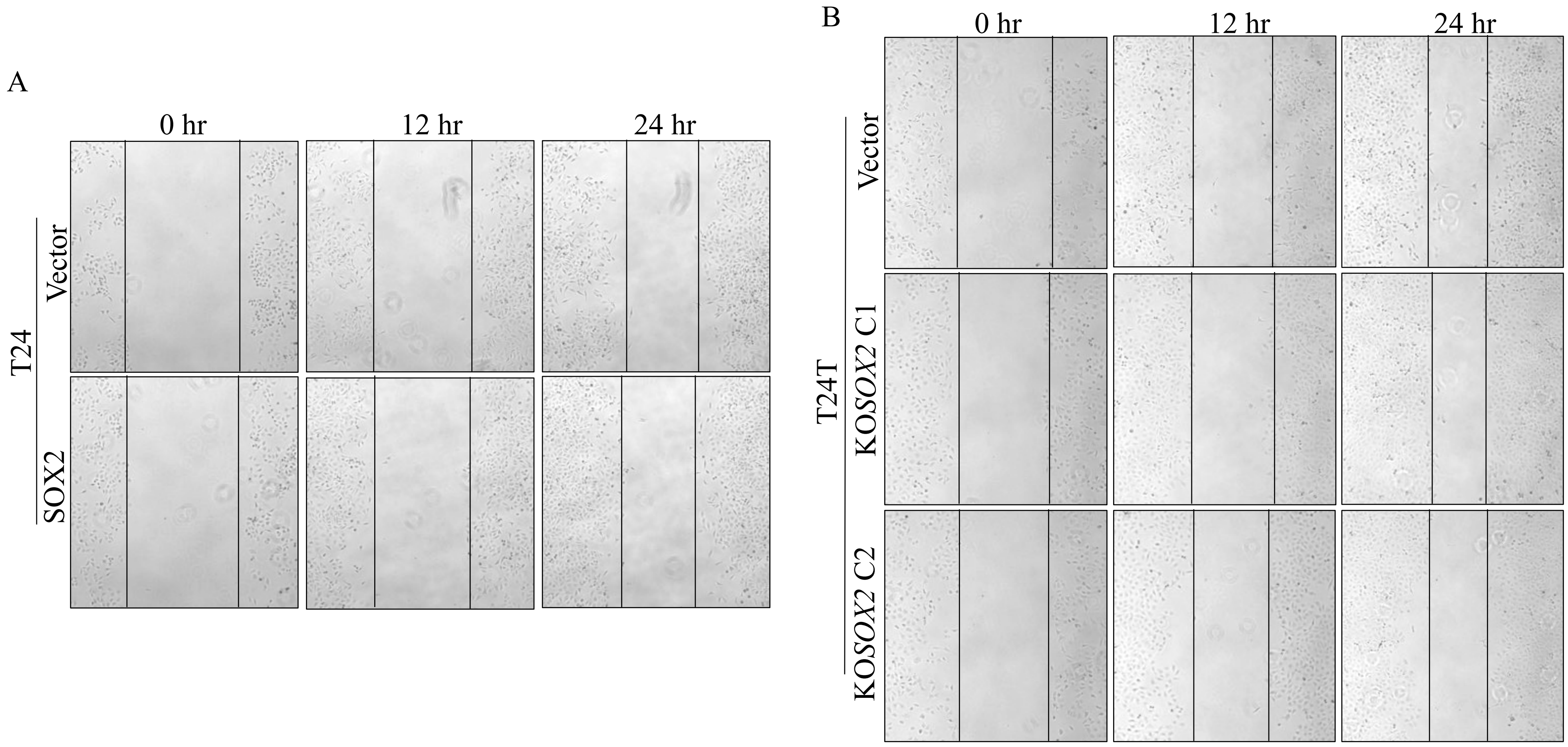

Figure S1. SOX2 does not regulate cell migration. Cell migration was determined by wound healing assays at the indicated times between T24(Vector) vs. T24(SOX2) (A), and T24T(Vector) vs. T24T(KO*SOX2* C1), T24T(KO*SOX2* C2) (B)

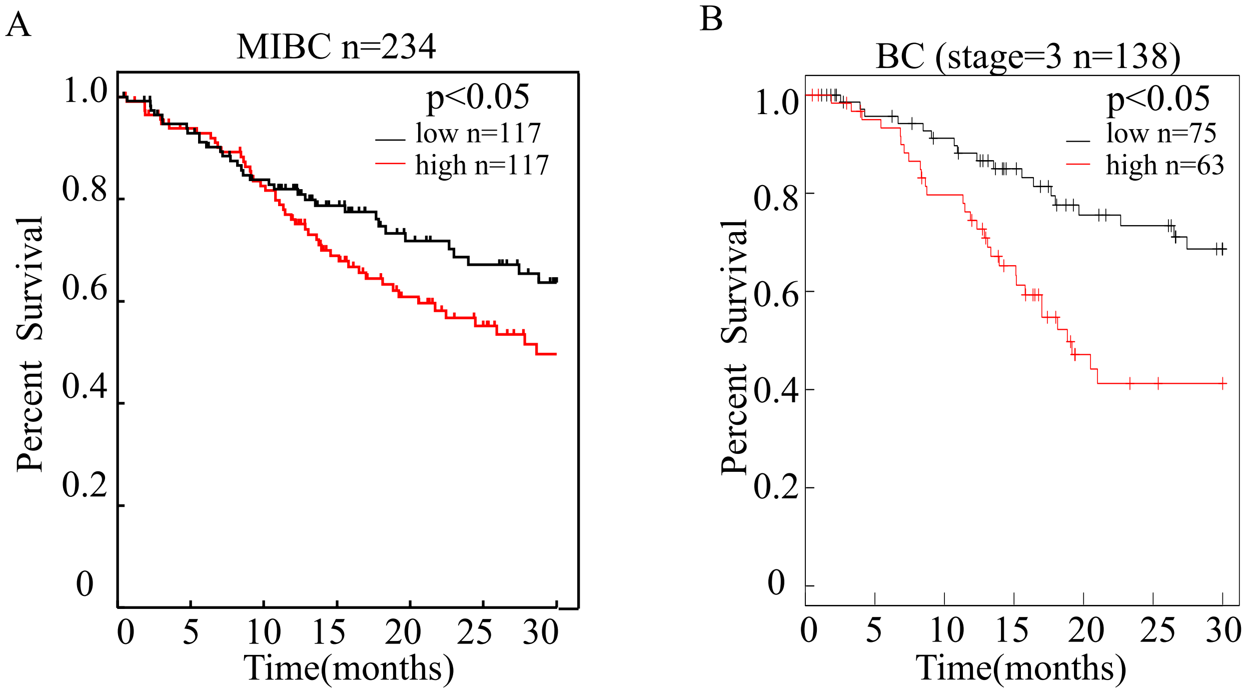

Figure S2. Kaplan-Meier estimation of SOX2 levels related overall survival (OS) in MIBC patients and stage 3 BC patients from the TCGA Database. Kaplan-Meier survival curves for MIBC patients with high or low expression of SOX2 (A). Kaplan-Meier survival curves for stage 3 BC patients with high or low expression of SOX2 (B). Low expression group: BC patients with lower SOX2 expression than the average level of all patients; high expression group: BC patients with higher SOX2 expression than the average level of all patients.

Xie Q. et al. Figure S3

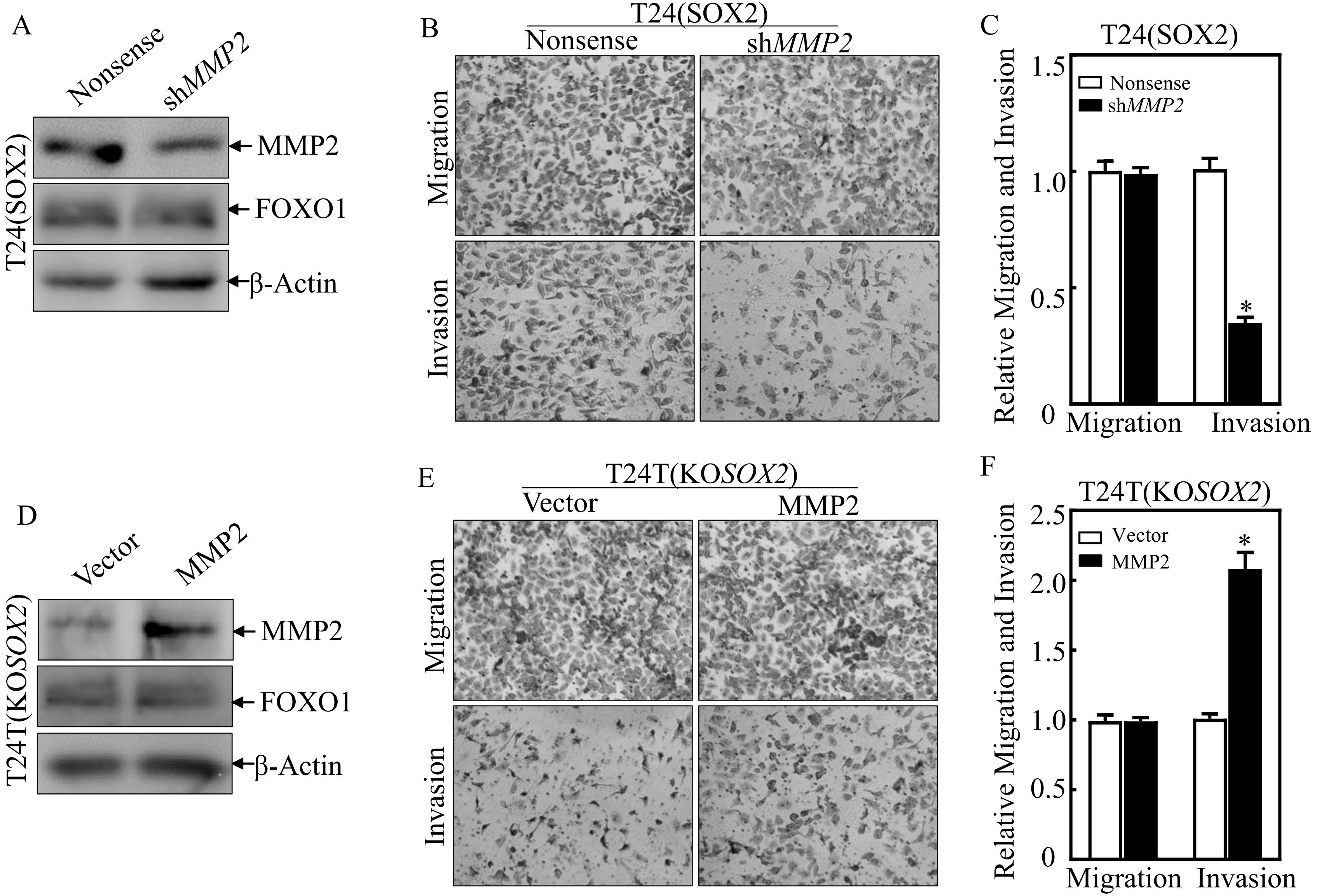

Figure S3. knockdown or ectopic expression of MMP2 on invasion ability. (A, D) The indicated cells were seeded into 6-well plates. The cells were extracted upon the cell density reaching 80–90%, and the cell extracts were subjected to Western blot for determination of protein expression as indicated. β-Actin was used as a protein loading control. **(B, C, E, F)** Invasion abilities of T24(SOX2/Nonsense) *vs* T24(SOX2/sh*MMP2*) cells and T24T(KO*SOX2*/Vector) *vs* T24T(KO*SOX2*/MMP2) were determined using BD BioCoat™ Matrigel™ Invasion Chamber. The asterisk (*) indicates a significant difference in invasion abilities in comparison to their corresponding vector control transfectants ($p<0.05$). The bars are presented as the mean ± SD from three independent experiments.

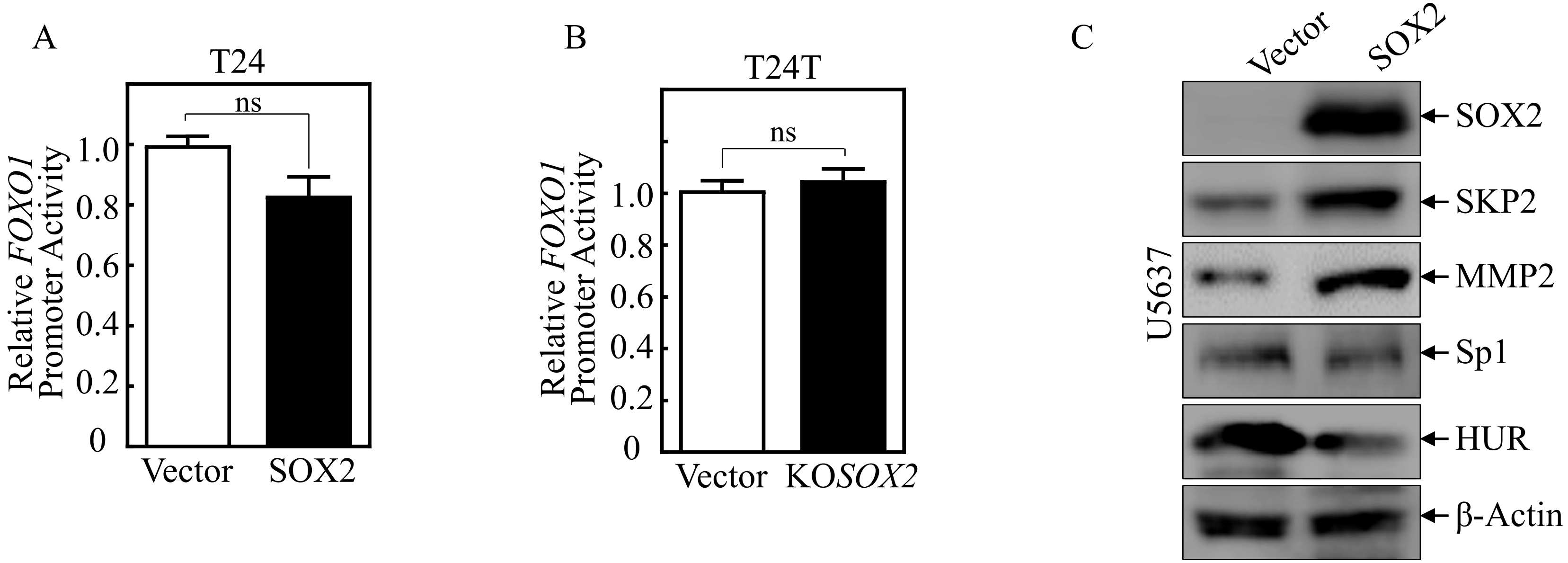

Figure S4. Effects of overexpression or KO*SOX2* on FOXO1 promoter activity. (A, B) The indicated cells were transfected with FOXO1 promoter-driven luciferase reporter together with pRL-TK. The transfectants were seeded into 96-well plates and then subjected to determine FOXO1 promoter activity by measuring luciferase activity. pRL-TK was used as an internal control to normalize transfection efficiency. Each bar indicates the mean ± SD from three replicate assays. **(C)** The indicated cells were seeded into 6-well plates. The cells were extracted upon the cell density reaching 80–90%, and the cell extracts were subjected to Western blot for determination of protein expression as indicated. β-Actin was used as a protein loading control. ns: not significant.

Xie Q. et al. Figure S5

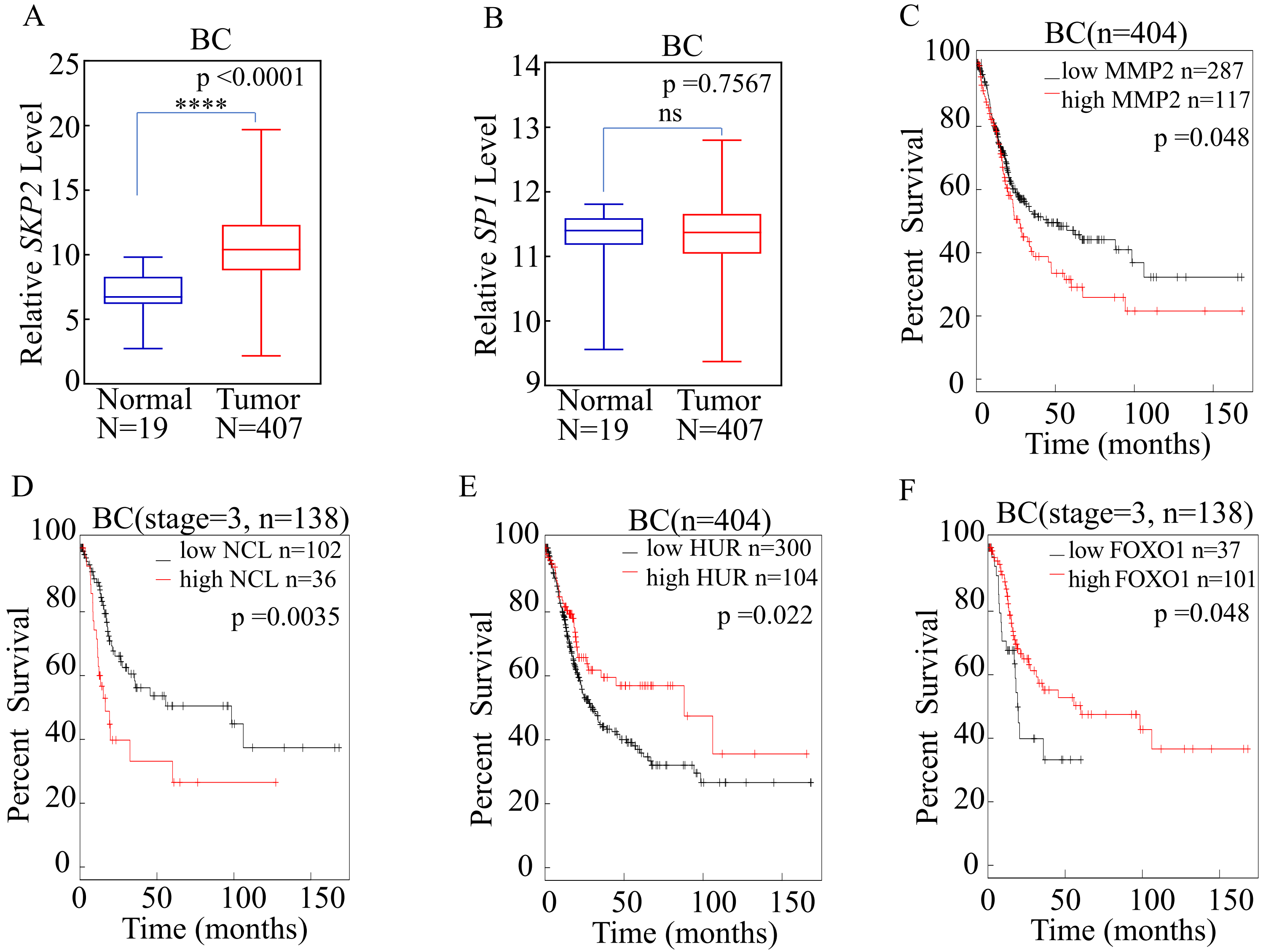

Figure S5. The expression level of SKP2 and SP1 and Kaplan-Meier estimation of MMP2, HUR, NCL and FOXO1 levels related overall survival (OS) in BC patients from the TCGA database. The expression level of SKP2 and SP1 in human bladder tumor vs. normal bladder tissues by RNA-Seq from the TCGA Database (A & B). Kaplan-Meier estimation of MMP2, HUR, NCL and FOXO1 levels related overall survival (OS) in BC patients from the TCGA Database (C-F). **** p<0.0001, ns: not significant.

Xie Q. et al. Figure S6

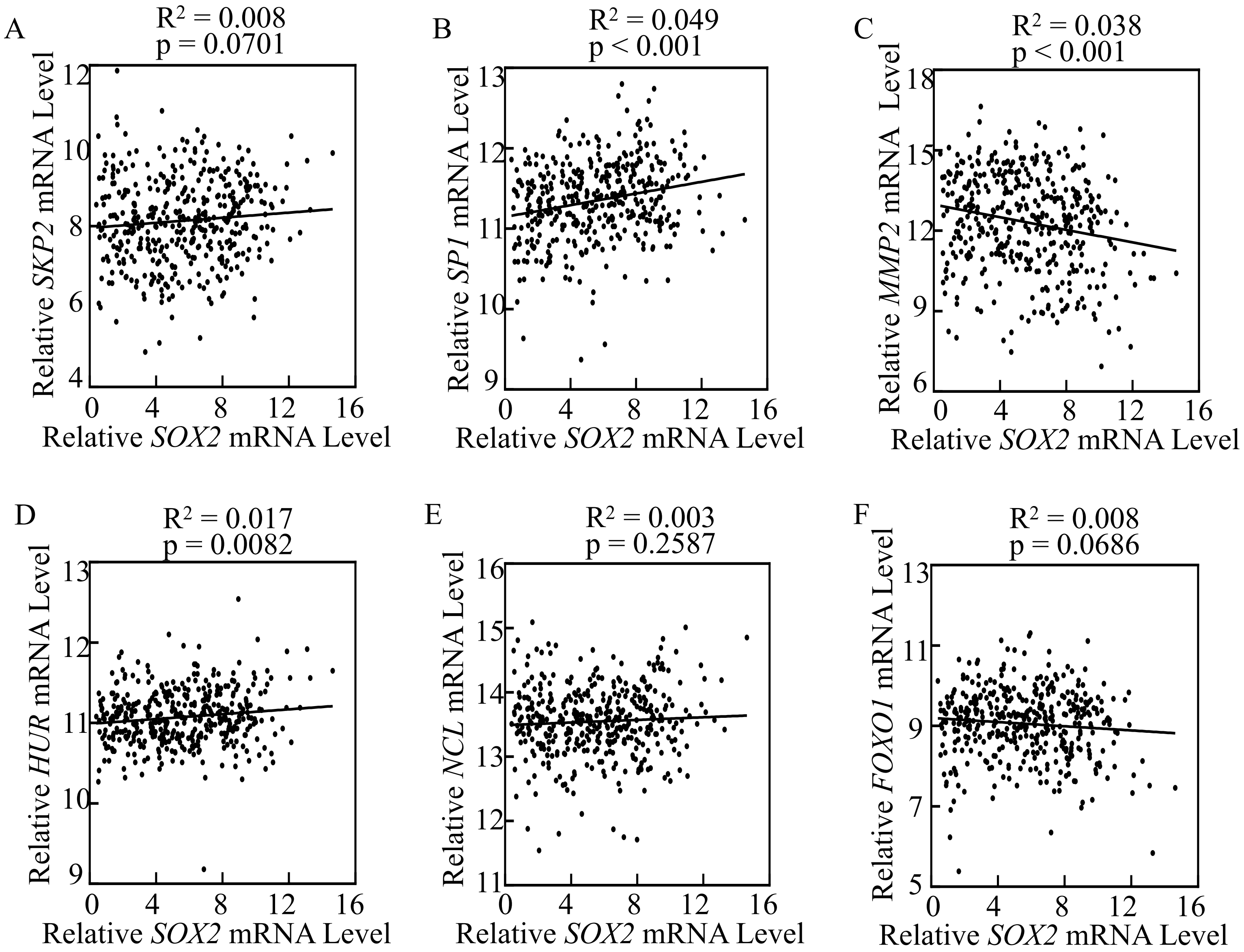

Figure S6. The gene expression correlation between SOX2 and other related gene expression, including SKP2, SP1, MMP2, HUR, NCL and FOXO1. The expression correlation between SOX2 and SKP2 (A), SP1 (B), MMP2 (C), HUR (D), NCL (E), FOXO1 (F).

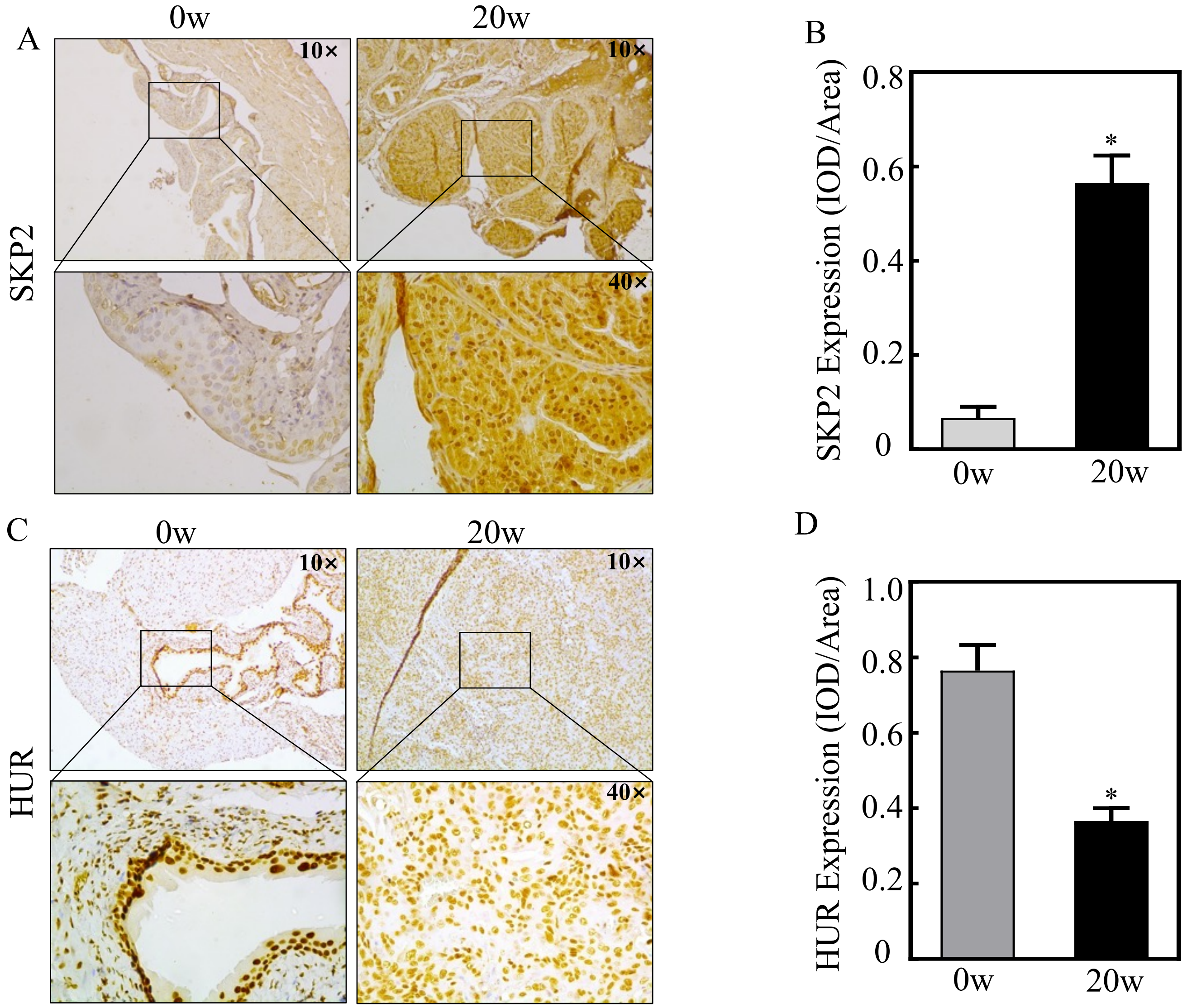

Figure S7. SKP2 was up-regulated and HUR was down-regulated in mouse BC tissues. (A - D) Mouse bladder tissues (n=15) with BBN treatment were collected for IHC staining to evaluate SKP2 and HUR protein expression. The representative images of each tissue were captured as described in the "Materials and Methods" section. SKP2 and HUR protein expression levels were analyzed by calculating the integrated IOD/area using Image-Pro Plus version 6.0. The results for each group were expressed as mean ± SD. Symbol (*) indicates a significant difference between 0 week control group and BBN-treated 20 weeks group (p<0.05).
